# H_2_, CO_2_, and CH_4_ Adsorption Potential of Kerogen as a Function of Pressure, Temperature, and Maturity

**DOI:** 10.3390/ijms232112767

**Published:** 2022-10-23

**Authors:** Arshad Raza, Mohamed Mahmoud, Saad Alafnan, Muhammad Arif, Guenther Glatz

**Affiliations:** 1 Department of Petroleum Engineering, King Fahd University of Petroleum & Minerals (KFUPM), Dhahran 31261, Saudi Arabia; 2 Department of Petroleum Engineering, Khalifa University, Abu Dhabi 127788, United Arab Emirates

**Keywords:** hydrogen, carbon dioxide, methane, storage, kerogen, adsorption, molecular simulation

## Abstract

We performed molecular dynamics simulation to elucidate the adsorption behavior of hydrogen (H_2_), carbon dioxide (CO_2_), and methane (CH_4_) on four sub-models of type II kerogens (organic matter) of varying thermal maturities over a wide range of pressures (2.75 to 20 MPa) and temperatures (323 to 423 K). The adsorption capacity was directly correlated with pressure but indirectly correlated with temperature, regardless of the kerogen or gas type. The maximum adsorption capacity was 10.6 mmol/g for the CO_2_, 7.5 mmol/g for CH_4_, and 3.7 mmol/g for the H_2_ in overmature kerogen at 20 MPa and 323 K. In all kerogens, adsorption followed the trend CO_2_ > CH_4_ > H_2_ attributed to the larger molecular size of CO_2_, which increased its affinity toward the kerogen. In addition, the adsorption capacity was directly associated with maturity and carbon content. This behavior can be attributed to a specific functional group, i.e., H, O, N, or S, and an increase in the effective pore volume, as both are correlated with organic matter maturity, which is directly proportional to the adsorption capacity. With the increase in carbon content from 40% to 80%, the adsorption capacity increased from 2.4 to 3.0 mmol/g for H_2_, 7.7 to 9.5 mmol/g for CO_2_, and 4.7 to 6.3 mmol/g for CH_4_ at 15 MPa and 323 K. With the increase in micropores, the porosity increased, and thus II-D offered the maximum adsorption capacity and the minimum II-A kerogen. For example, at a fixed pressure (20 MPa) and temperature (373 K), the CO_2_ adsorption capacity for type II-A kerogen was 7.3 mmol/g, while type II-D adsorbed 8.9 mmol/g at the same conditions. Kerogen porosity and the respective adsorption capacities of all gases followed the order II-D > II-C > II-B > II-A, suggesting a direct correlation between the adsorption capacity and kerogen porosity. These findings thus serve as a preliminary dataset on the gas adsorption affinity of the organic-rich shale reservoirs and have potential implications for CO_2_ and H_2_ storage in organic-rich formations.

## 1. Introduction

Global warming is evident and has led to significant adverse impacts on various Earth systems around the globe [1] due to the burning of fossil fuels and the continuous release of greenhouse gases (GHG) [2,3]. In this context, the Paris Agreement was signed by 195 parties to limit global warming to 2 °C in an attempt to maintain it below 1.5 °C by 2050 [4]. Two main strategies for reducing atmospheric concentrations of CO_2_ are negative emissions technology and/or switching to low- or zero-carbon fuel sources [5,6]. First, carbon capture and storage (CSS) technology is considered effective negative emission technology that is a necessity, not an option, and can cut 19% of global CO_2_ emissions by 2050 [7]. CCS involves capturing CO_2_ from stationary sources, e.g., fossil fuel power plants, fuel processing plants, and other industrial plants, particularly iron, steel, cement, and bulk chemical plants. Then, the captured CO_2_ is transported through pipelines or ships for storage in underground geological formations, such as saline aquifers, depleted reservoirs, and coal seams [8,9,10,11]. Renewable energy sources are low- or zero-carbon fuel sources, which have been recognized as an effective solution to mitigate the associated global warming concern [12,13]. This is evident from large-scale renewable energy projects to generate electricity and large-scale CO_2_ sequestration projects across the globe (e.g., Europe [14] and the Gulf region [15]). According to the World Energy Outlook, renewable-based energy sources could supply 30% of the total global energy by 2040 [13]. In this context, while CO_2_ geo-sequestration remains a major interest [3,16,17,18,19,20], the use of H_2_ as clean energy and its storage, which originated a few decades ago [21,22], has recently gained notable attention too [23,24,25]. The key benefit of hydrogen is its low carbon footprint, which could revolutionize the global energy outlook as a fuel [26]; however, specific challenges are associated with the low density and high diffusivity of H_2_ [27]. Although several initiatives have been taken to mitigate serious climate change consequences, more efforts are still needed to address global warming.

Subsurface geologic formations, i.e., depleted hydrocarbon reservoirs and saline aquifers, have been explored for large-scale CO_2_ [28,29,30,31,32,33] as well as hydrogen (H_2_) storage [34]. Recently, coal seams [26] and basaltic rocks [35] have also been investigated for their H_2_ storage potential, albeit at a lab scale. Furthermore, shale rocks have been evaluated for large-scale CO_2_ storage via adsorption trapping [36,37,38], while a recent study investigated H_2_ storage in inactive shale [39]. While the idea of underground hydrogen storage (UHS) focuses on energy storage, CO_2_ flooding in a medium comprised of shale and coal offers double advantages, i.e., it addresses carbon capture and storage (CCS) and enhances methane (CH_4_) recovery [34,40,41,42].

Shale rock is a clastic sedimentary rock in origin with fine-grained clastic sedimentary rock and exists in many types and maturities [40,43]. Shale microstructure is formulated by organic kerogen, inorganic minerals, and the cleat network [43,44]. Clay-based source rock and clastic sedimentary formations (e.g., fine-grained limestones) contain abundant kerogens, possibly 80–99% [45,46]. In kerogen-rich shales, gas can adsorb onto the rock surface and absorb onto and within the pore space of the organic matter as well as some clay minerals [47]. Particularly, the shale surface facilitates adsorption as a result of weak interaction forces (van der Waals and electrostatic), as evidenced in classical observations [48]. Adsorption is typically affected by pressure, temperature, and surface roughness [47]. It is well known that sorption (adsorption and absorption) is a reversible process due to the absence of covalent bonds between the adsorbates (fluids) and the adsorbent (rock surface) [47]. CO_2_ has a high adsorption tendency to organic matter [49]. The adsorption capacities of CO_2_ and CH_4_ in shale are affected by pressure, temperature, and maturity [47,50], e.g., adsorption rises with pressure and maturity and declines with temperature, while the adsorption trend exhibits mainly type 1. It is noteworthy that adsorption/desorption assists in estimating the storage capability of gas in shale, whereas kinetic diffusion determines the fluid flow in the porous media of shale [47]. This adsorption capacity is exceedingly challenging and influenced by subsurface operating conditions, i.e., pressure and temperature [51]; organic matter richness and surface area [47]; thermal maturity [52]; kerogen type, i.e., type I–III [53]; and mineral type, which are montmorillonite, illite, kaolinite, and chlorite [54]. Published experimental data suggest that shale exhibits a significant capacity to adsorb different gases, such as CO_2_ and methane [50,55,56], arguably due to favorable CO_2_-wetting characteristics [57]. This observation is also in agreement with molecular-level quantification of CO_2_ and CH_4_ adsorption on shale [40,52,58]. Generally, methane exhibits a 10–30 times lower adsorption capacity in shale than in coal [59], while CO_2_ can adsorb 5–10 times more than CH_4_ [50].

A range of experimental techniques (e.g., mercury intrusion capillary pressure (MICP) and neutron scattering) are used to determine adsorption potential and pore size distribution [60], while the molecular dynamics (MD) modeling approach describes gas adsorption in kerogen models, which act as an assembly of molecules [61]. Kerogen is composed of an intrinsically complex amorphous carbon network with pore sizes ranging from angstrom to micrometer sizes [62]. From a fundamental geochemical point of view, shale kerogen demonstrates a range of physicochemical features, including but not limited to kerogen porosity, the maturity indicator, the hydrogen-to-carbon ratio, the oxygen-to-carbon ratio, and the aromatic/aliphatic ratio [63]. Thus, a cross-plot of these features in a classic van Krevelen diagram describes the kerogen of different deposition origins, including type I (lacustrine), type II (marine), type III (terrestrial), and type IV (originating from residues), and the molecular models of these kerogens have been investigated [61]. 

Out of all kerogen models, type II kerogen is known to be the main origin of unconventional (shale) gas play [52,64]. Type II kerogen is further classified into four categories—immature, top of oil window, middle/end-of-oil window, and overmature—and each model is characterized by its respective maturity indicators [52]. Thus, a few previous studies [52,65,66] have investigated the gas adsorption potential of these four sub-models of type II kerogen. While these investigations have revealed the gas adsorption potential of kerogen, the impact of kerogen maturity on the gas adsorption potential of shales requires further investigation. Moreover, while the area of underground hydrogen storage is advancing, there is still limited research on H_2_ storage via adsorption trapping. Only a few studies have reported the H_2_ adsorption potential of rocks, e.g., H_2_ adsorption on clay [51] and H_2_ adsorption in coals [26], and these studies have reported a significant potential for hydrogen adsorption that was sensitive to pressure and temperature. However, the effect of organic matter residing in shale and its maturity has not been evaluated yet for H_2_ sorption potential. Moreover, a comparison of H_2_ sorption capacity with CO_2_ and CH_4_ has not been reported, but it is, nevertheless, of great importance in terms of exploring shale as a potential medium for H_2_ storage. 

This study, therefore, investigated the adsorption behavior of hydrogen (H_2_), carbon dioxide (CO_2_), and methane (CH_4_) on four kerogen (organic matter) samples of varying maturity (A < B < C < D) over a wide regime of pressures (2.75 to 20 MPa) and temperatures (323 to 423 K). The molecular simulations were performed using the grand-canonical Monte Carlo (GCMC) simulation module to mimic subsurface conditions. We also correlated the adsorption capacity of CO_2_, CH_4_, and H_2_ to a range of kerogen structural parameters, i.e., atom ratios, % of aromatic carbon, and oxygen atoms. These results provide a fundamental understanding of gas storage in shales and particularly underpin the large-scale CO_2_ and hydrogen storage potential in organic-rich shale reservoirs and associated decarbonization strategies.

## 2. Result and Discussion

### 2.1. Gases Adsorption Behavior against Pressure

**Figure 1:**
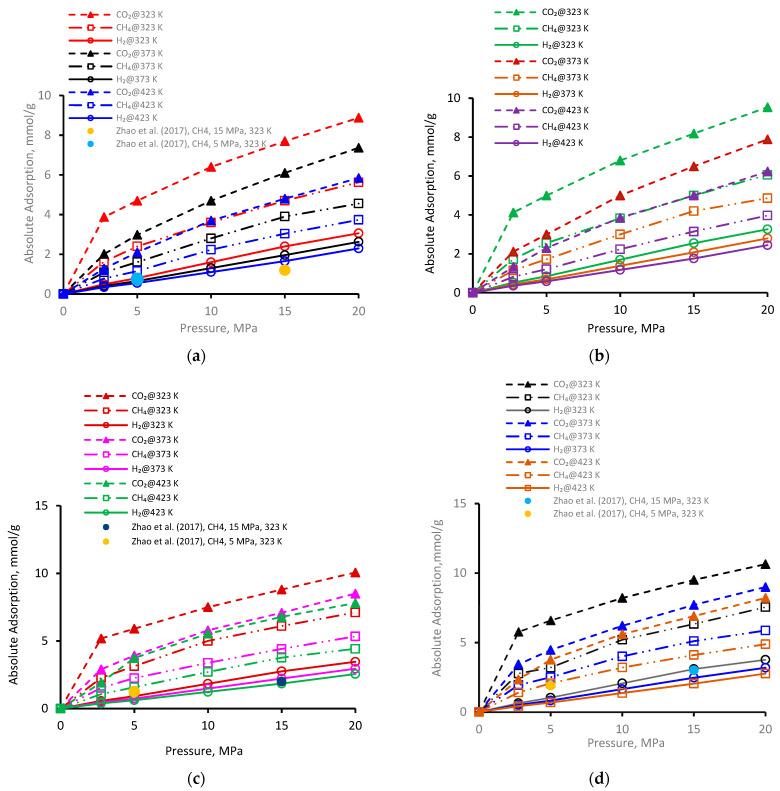
Gas adsorption behavior versus pressure at different temperatures on kerogen [52]; (**a**) II-A, (**b**) II-B, (**c**) II-C, (**d**) II-D.

The adsorption of gases (CO_2_, CH_4_, and H_2_) was investigated for a broad range of pressure regimes (2.75 to 20 MPa) on four kerogen structures (Figure 1a–d). Clearly, gas adsorption on all types of kerogen structures increased with increasing pressure (Figure 1a). Commonly, at a particular pressure and temperature, the adsorption of three gases followed the order CO_2_ > CH_4_ > H_2_. As an example, for the pressure increment from 2.75 MPa to 20 MPa at 323 K, CO_2_ adsorption on II-A kerogen increased nearly 2.2 times (from 3.8 mmol/g to 8.8 mmol/g), 3.4 times (1.6–5.6 mmol/g) in case of CH_4_, and 6.2 times (0.49–3.0 mmol/g) in case of H_2_ (Figure 1a). Furthermore, the observed adsorption behavior was likely to plateau beyond pressure 20 MPa. The highest adsorption capacity of CO_2_ confirmed its tendency to desorb CH_4_ during a coupled enhanced methane recovery and CO_2_ sequestration in organic-rich shales, consistent with previous observations [38,50]. When the H_2_ adsorption was lowest and thus H_2_ could not desorb CH_4_ and CO_2_, the observations suggested the potential for H_2_ storage via adsorption trapping in shale formations.

Furthermore, for the immature type II-A kerogen, the highest recorded adsorption capacity was 8.8 mmol/g for CO_2_ at 323 K and 20 MPa (but less than overmature II-D kerogen). Gas adsorption in II-A kerogen was directly correlated with pressure, which can be attributed to the enhanced van der Waals and electrostatic interactions between the gas molecules (adsorbates) and the surface (adsorbent) at a higher pressure that lead to physisorption. 

Similar trends were noted for gas adsorption on II-B (Figure 1b), II-C (Figure 1c), and II-D (Figure 1d), i.e., the increase in adsorption with the increase in pressure. At a low-pressure, the relative adsorption capacities were higher than in the high-pressure range, which can be attributed to the fact that the highest adsorption energy is found in the smallest pores at low pressure at first and then advances toward the larger pores with increased pressure, which in turn decreases the isosteric heat of adsorption [67]. This observation is an indication of pore filling by physisorption in microporous material, as first recommended by Dubinin [68]—a small increase in adsorption with a further increase in pressure until equilibrium is established. As a comparison, in the literature data from Zhao et al. [37] for similar kerogen (without nanopores), the two data points for II-A, II-C, and II-D (at 5 MPa and 323 K and 10 MPa and 323 K, plotted in Figure 1a,c,d) indicated a relatively lower adsorption capacity under similar conditions compared with our results. This underestimation could be attributed to the absence of nanopores with major control over the adsorption behavior, and thus the presence of nanopores (as in our models) suggests greater adsorption capacity. 

**Figure 2:**
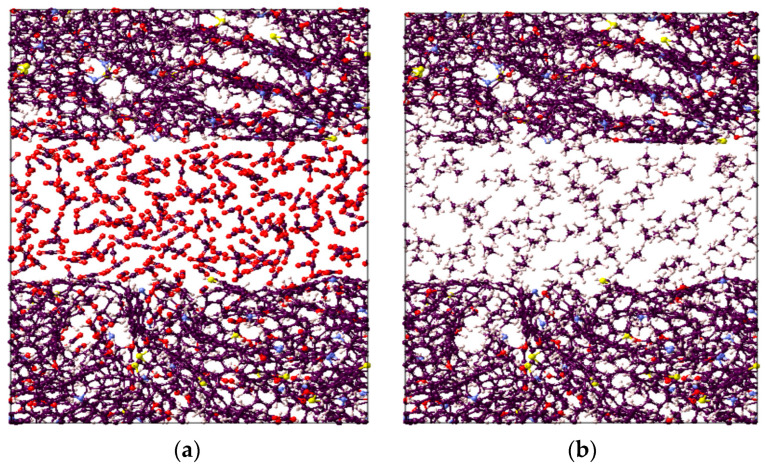

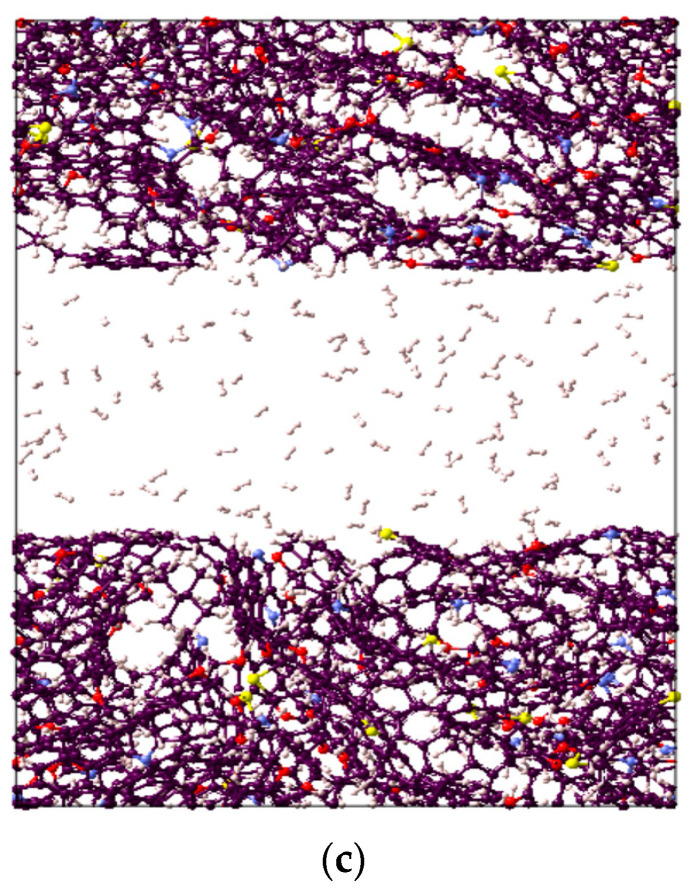
Visual representation of the constructed molecular model for active spots available in II-D overmature-type kerogen for (**a**) CO_2_, (**b**) CH_4_, and (**c**) H_2_ absorption within the structure and adsorption on the surfaces.

It is worthwhile to note that the overall highest adsorption capacity was noted to be 10.6 mmol/g for CO_2_ in immature II-D kerogen at 20 MPa and 323 K (Figure 1d). To visualize this better, a nanopore layer model of type II-D kerogen is shown (Figure 2), indicating the adsorption behavior of all gases, and a large cluster of CO_2_ molecules is evident (Figure 2). In addition, all kerogen structures depicted mainly type I adsorption behavior for gases, consistent with recent findings [52,69,70]. This observation is also in agreement with CO_2_ and CH_4_ adsorptions on coal [71]. In the case of H_2_ adsorption, however, no attempt on kerogen is available to compare with the results of this study. Note, however, that here, the kerogen has a strong affinity for CO_2_, i.e., ~1.7 times than CH_4_ and 4.5 times more than H_2_, which can be attributed to kerogen functional groups, which have a notable effect on the adsorption of CO_2_, CH_4_, and H_2_ because of their remarkable adsorption energy for CO_2_ over CH_4_ and H_2_ [69]. It can be noted that kerogens exhibited a small deviation from a type I adsorption behavior for H_2_ gas. This particular behavior can be credited to the small molecular mass of H_2_ (2.016 g/mole [6]) or low density, which promotes the weak intermolecular interaction of H_2_ with organic matter and affects adsorption thermodynamics, and the associated bonding between adsorbate and adsorbent is a strong function of the density of a gas [47]. 

Hence, in short, the results advise a significant capability for gas disposal in underground organic-rich (kerogen) shale formations via adsorption trapping, especially for overmature kerogens. Furthermore, the observed adsorption isotherms are of great importance to be used as input in reservoir-scale assessment to investigate organic-rich porous media [71].

### 2.2. Gases Adsorption Behavior against Temperature

**Figure 3:**
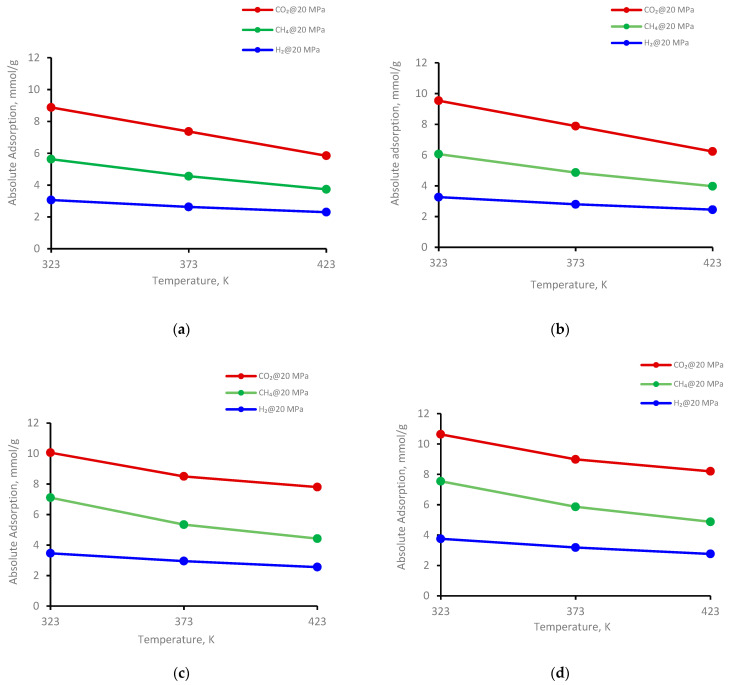
Gases adsorption trend versus temperature at 20 MPa on kerogen; (**a**) II-A, (**b**) II-B, (**c**) II-C, (**d**) II-D.

The adsorption behavior on four kerogen structures of the gases (CO_2_, CH_4_, and H_2_) was evaluated at 323 K, 373 K, and 423 K (Figure 3). Clearly, adsorption decreased with increasing temperature for all kerogen structures. For example, at a fixed pressure of 20 MPa for II-A kerogen, the adsorbed amount of CO_2_, decreased from 8.8 mmol/g to 5.8 mmol/g (i.e., a 66% reduction) when the temperature of the system was elevated from 323 K to 423 K (Figure 3a), indicating a prominent decline. Similar trends were evident for CH_4_ and H_2_, i.e., a decline from 5.6 mmol/g to 3.7 mmol/g (↓66%) for CH_4_ and from 3.0 to 2.9 mmol/g (↓75%) for H_2_ for the same temperature increment (Figure 3a). At the same temperature range at a fixed pressure of 20 MPa, the adsorption capacity of the gases in other kerogens (II-B, II-C, and II-D) decreased with similar trends, as noted in II-D (Figure 3b–d and Table 1). Thus, the adsorption capacity is sensitive to temperature, and the low-temperature shale gas reservoir demonstrated greater gas storage potential than the high-temperature shale formation. This phenomenon was also observed in shale adsorption tests [52,72,73]. 

The temperature directly caused the gas molecules to leave the adsorption site by high kinetic energy and escape to the bulk-free phase, i.e., a reduction in the adsorbed phase density with increasing temperature [26]. Consequently, the amount of adsorbed gas decreased with increasing temperature [73]. Moreover, the wetting behavior of shale could be another factor responsible for the lower adsorption of gases at higher temperatures. This is evident from the reduction in the water’s advancing and receding contact angles with increasing temperature on shale surfaces [74] as well as coal samples [75], i.e., the samples showed less affinity toward CO_2_ at higher temperatures. However, further investigations are required to confirm these relationships. Thus, low-temperature shale appears to be most suitable for gas storage. 

### 2.3. Gases’ Adsorption Capacity versus Thermal Maturity

**Figure 4:**
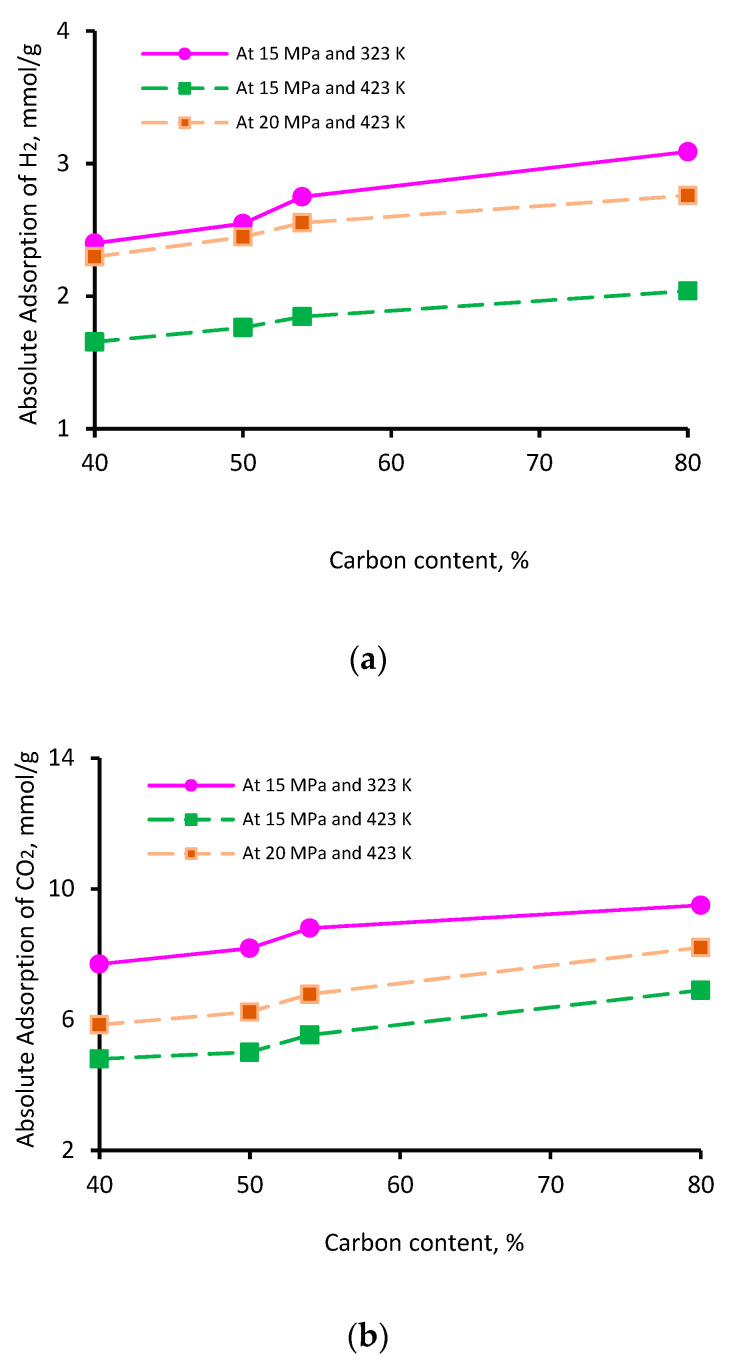

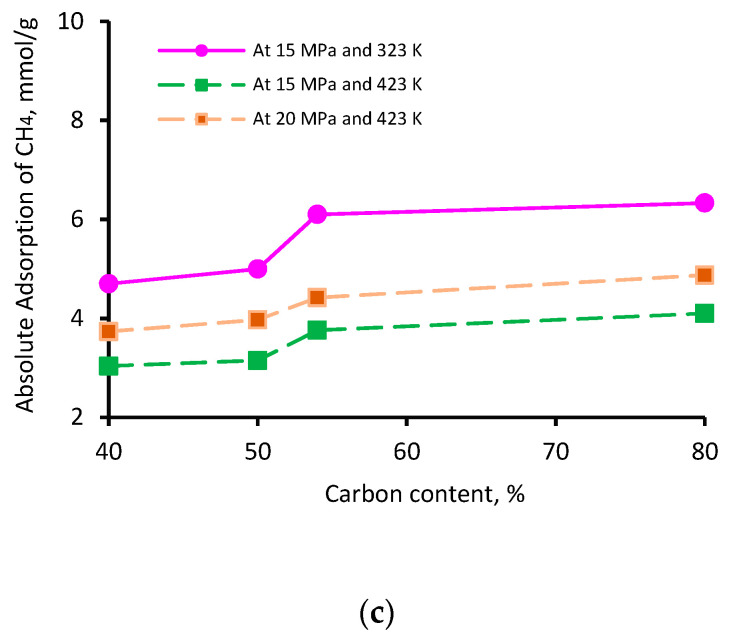
Gas adsorption (**a**) H_2_, (**b**) CO_2_, and (**c**) CH_4_ as a function of carbon content.

The thermal maturity of a shale sample is known to influence its gas storage capacity. While vitrinite reflectance (Vr) is typically used as an indicator of thermal maturity [76], here, we used the elementary analysis of the four kerogen structures to establish the influence of maturity; this is because this study was based on kerogen models as opposed to a physical shale sample. The percentage of aromatic fixed carbon (obtained from NMR [77]) was lowest in II-A kerogen (40%) and highest in II-D kerogen (80%). Similarly, the XPS analysis demonstrated a similar trend [77]—the lowest aromatic carbon in II-A (40%) and the highest in II-D kerogen (72%)—consistent with overmature kerogen. Thus, on the basis of these model parameters, kerogen maturity was in the order of II-D > II-C > II-B > II-A. The adsorption capacity of the three gases demonstrated a clear increase with increasing kerogen maturity. This behavior can be attributed to specific functional groups, i.e., H, O, N, and S, and an increase in effective pore volume, as both are correlated with organic matter maturity, which is proportional to adsorption capacities [69,78,79]. Typically, at a particular pressure and temperature, the adsorption capacity followed the trend II-A < II-B <II-C < II-D, and the adsorption capacity increased with increasing carbon content (Figure 4). With an increase in carbon content from 40% to 80%, the adsorption capacity increased from 2.4 to 3.0 mmol/g for H_2_ (Figure 4a), 7.7 to 9.5 mmol/g for CO_2_ (Figure 4b), and 4.7 to 6.3 mmol/g for CH_4_ (Figure 4c) at 15 MPa and 323 K. It is noteworthy to mention that the carbon content develops microporous characteristics in shale and contributes to the surface area and total pore volume. These factors can be attributed to a surge in absolute H_2_, CO_2_, and CH_4_ adsorption above 50% of the carbon content (which can be referred to as the critical carbon content).

Thus, it was confirmed that highly mature kerogen mainly adsorbs more gas, preferably CO_2_ over CH_4_ and H_2,_ while immature kerogen (II-A: carbon content = 40%) showed the lowest adsorption capacity. These results suggest a positive correlation between kerogen maturity and the adsorption capacity of gases. These observations are consistent with previous findings on CO_2_ and CH_4_ adsorption onto shale surfaces [52,53,78]. A recent investigation by Arif et al. [26] also found a consistent increase in H_2_ adsorption in coals with increasing carbon content [26]. 

### 2.4. Effect of Kerogen Porosity on Adsorption

The pore size distribution of the samples investigated here suggests that II-D exhibited the largest porosity (= 0.144), followed by II-C (= 0.075), II-B (= 0.073), and II-A (= 0.056), in descending order [80], i.e., the porosity increased with increasing thermal maturity from II-A to II-D kerogens. This observation is in agreement with a previous study [81]. Accordingly, with an increase of micropores, porosity was increased, and thus II-D offered the maximum adsorption capacity, and II-A kerogen offered the minimum. For example, at a fixed pressure (20 MPa) and temperature (373 K), the CO_2_ adsorption capacity of type II-A kerogen was 7.3 mmol/g, while type II-D adsorbed 8.9 mmol/g under the same conditions (Figure 1). Thus, a higher kerogen porosity promoted greater gas adsorption. Similar trends were evident for other gases (Figure 1). 

Previous studies have evaluated the impact of pore size on gas adsorption potential in kerogen structures and shale and have noted that the adsorption capacity is indirectly correlated with the pore size increases [82,83,84,85,86]. The reason behind this could be limited adsorption heat and interaction energy with increased pore size [83]. This observation underpins the phenomenon of adsorption via enhanced specific surface area [86,87]. Notably, smaller micropores (<2 nm), evident in all kerogens, can provide a greater surface area and thus greater gas adsorption capacity [47]. However, the average pore sizes were almost the same in all kerogens.

Our results are in alignment with past studies [80,88,89], i.e., adsorption is sensitive to porosity. This can be attributed to the structural transformation, increased number of pores, and active sorption sites from immature to overmature shale that provide strong interaction sites to gases for adsorption. Small pore volumes were dominant in kerogens II-A and II-B, while large pore volumes contributed to kerogen II-C and II-D [78]. Thus, porosity’s effect on the adsorption is clearly evident—a direct correlation was observed between kerogen porosity and adsorption capacity. Like the porosity effect, it is vital to extend this work to the effect of fracture permeability [90] on adsorption.

## 3. Materials and Methods

### 3.1. Kerogen Structure

**Figure 5:**
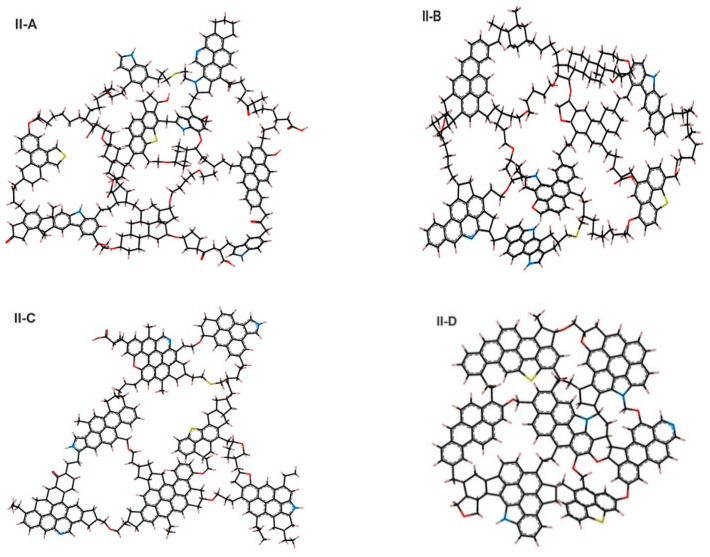
Six kerogen units developed by Ungerer et al. [91], representative of real kerogen macromolecules before structural optimization. A single atomic unit of kerogen (also known as macromolecule) comprised of carbon (black), oxygen (red), sulfur (yellow), nitrogen (blue), and hydrogen (gray).

We used four kerogen molecular models of varying maturity, i.e., immature (type II-A), top of oil window (type II-B), middle/end of oil window (type II-C), and postmature kerogen (type II-D) were considered (Figure 5). These models are similar to those of Ungerer et al. [91], which were based on the analytical data corresponding to the work of Kelemen et al. [92]. The chemical compositions of the four kerogen models were *C_252_H_294_O_24_N_6_S_3_*, *C_234_H_263_O_14_N_5_S_2_*, *C_242_H_219_O_13_N_5_S_2_*, and *C_175_H_102_O_9_N_4_S_2_*. Note that such kerogen models exist in the organic-rich shale (e.g., Duvernay marine).

The chemical composition and physical properties of kerogen structures were in good agreement with the experimental data (e.g., X-ray and Nuclear Magnetic Resonance) [92], and the percentage of aromatic fixed carbon demonstrated a trend: the lowest aromatic carbon in II-A and highest in II-D kerogen. Thus, the kerogen maturity and porosity were in the order of II-D_(overmature)_ > II-C_(Middle and End Oil Window)_ > II-B_(Top of Oil Window)_ > II-A_(immature)_. The pores size distribution demonstrated that II-D contained the largest micropores, followed by II-C, II-B, and II-A in descending order.

**Figure 6:**
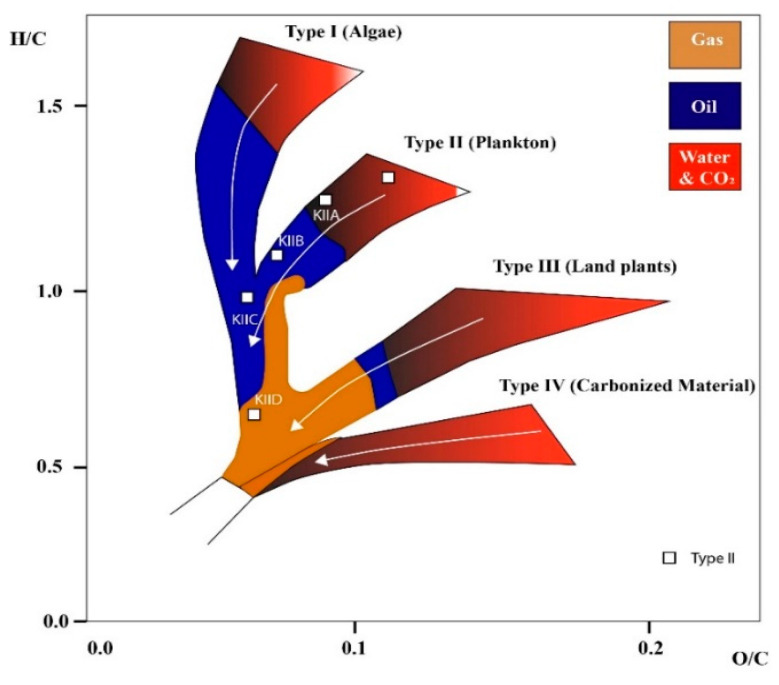
Kerogen structure classification indicated as increasing order of maturity (modified from [94]). White arrow shows increased level of maturity.

Furthermore, immature kerogen had higher O/C and H/C ratios and lower aromaticity than the oil window and post-mature kerogens, while the key element of the structures was that the maturity increased as follows: II-A < II-B < II-C < II-D (see Table 2), which is plotted on Van Krevelen diagram illustrated in Figure 6. Specifically, II-D had the largest micropores, as evidenced by its high value of porosity compared with other considered kerogen types. Specifically, II-D had the largest micropores, as evidenced by its high value of porosity compared with other considered kerogen types.

### 3.2. Kerogen Model Construction 

A few previous MD simulation studies [52,58] have been carried out to develop the condensed kerogen using a large-scale atomic/molecular massively parallel simulator (LAMMPS) [80]. Polymer-consistent forcefield plus (pcff++) was used in molecular dynamics [93]; it describes atom dispersion/repulsion (Lennard–Jones potential 6–9), intermolecular, and electrostatic interactions and has been recognized as a reliable forcefield for thermodynamic characteristics [77,95].

**Figure 7:**
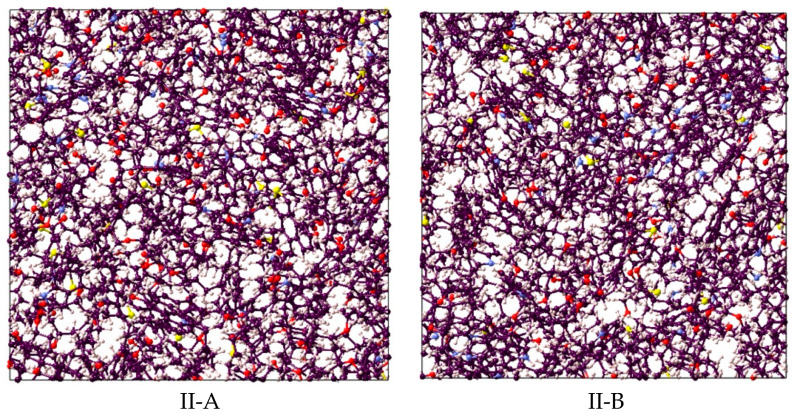

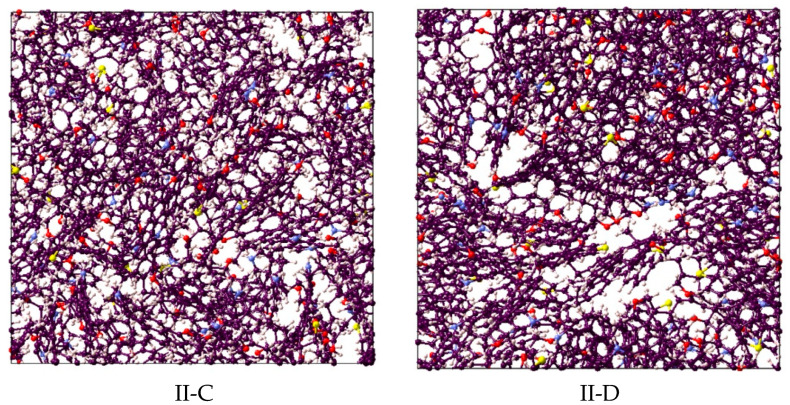
Final kerogen structure comprised of carbon (black), oxygen (red), sulfur (yellow), nitrogen (blue), and hydrogen (gray).

**Figure 8:**
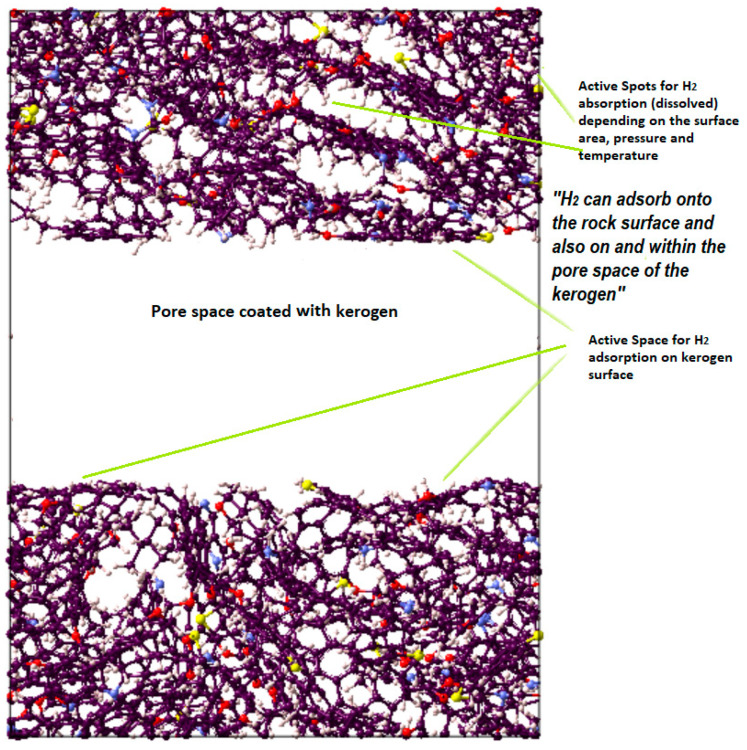
Visual representation of the molecular model for active spots available in II-D (overmature-type kerogen) H_2_ absorption within the structure and adsorption on the surfaces.

Kerogen structures of different maturities and types containing different units were used. A pressure of 20.7 MPa and a temperature of 350 K were considered to progress the simulation and were supposed to be representative of reservoir conditions. The MD simulation considered initialization (9.5 cutoff value and periodic boundary) and energy minimization (molecular positions and velocity proper configuration). Subsequently, the NVT (isochoric-isothermal) and NPT (isobaric-isothermal) simulations were run at 336 K for 250 ps and 200 ps, respectively. Through three continuous NPT steps, the temperature was gradually decreased from 350 K to 336 K to attain faster convergence of kerogen units, as depicted by the final structure in Figure 7. To study the adsorption on the surface of kerogen, a nanopore was created in kerogen to expose the pore space to host the gas (i.e., CO_2_, CH_4_, or H_2_), which led to adsorption on the surface. Unlike absorption, the adsorbed molecules do not penetrate the kerogen structure, and thus there was no chance of internal structural changes. Thus, the sum of adsorption and absorption, sorption, is equivalent to the total storage capacity. The kerogen model (e.g., II-D) used in this study is visualized in Figure 8; the other kerogen structure depicts a similar illustration of active space for adsorption. 

### 3.3. Simulation Detail

**Figure 9:**
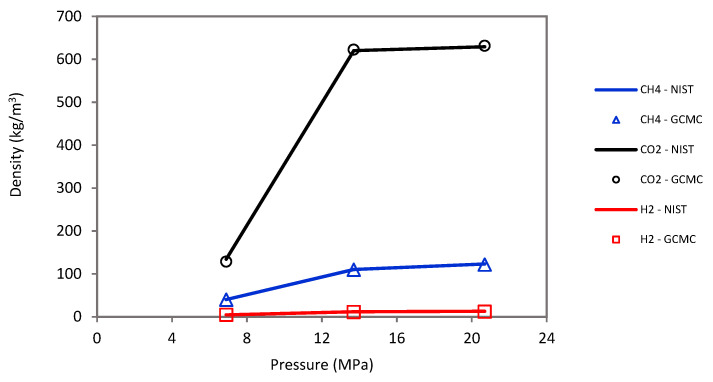
Molecular loading approach shows an acceptable density match between empty cell yields and experimental NIST data for CO_2_, CH_4_, and H_2_.

The sorption was simulated by the Monte Carlo (MC) technique using the grand-canonical Monte Carlo (GCMC) approach [52,69,94] under a wide range of pressures (2.75 to 20 MPa) and temperatures (323 to 423 K). Moreover, ASPEN software was used with the Peng–Robinson equation of state to calculate fugacity coefficients of host molecules (i.e., H_2_, CH_4_, and CO_2_) and converted into chemical potential in the GCMC simulation. Note that the host molecules were defined as united atoms. A molecular loading approach was further adopted to validate the molecular simulation, which showed an acceptable match between the empty cell yield density and the experimental NIST data for CO_2_, CH_4_, and H_2_ (see Figure 9). 

Interaction between kerogen and host molecules took place using the 6–9 LJ function (Equation (1)) with a grid spacing of 0.2 Å. For cross interactions, the Lorentz–Berthelot mixing rules (Equations (2) and (3)) were used. Upon the adsorption of gas molecules over kerogen up to a certain value, equilibrium was reached. The number of interactions was set at 0.35 million. At the end of the simulation, the total average adsorbed gas molecules were obtained as a function of chemical potential. Further explanation of the GCMC simulation procedure can be found in the literature [52,58].

(1) $$U_{LJ}=\epsilon \left[2{\left(\frac{\sigma }{r_{ij}}\right)}^{9}-3{\left(\frac{\sigma }{r_{ij}}\right)}^{6}\right]+\frac{q_{i}q_{j}}{4\pi \epsilon_{0}r_{ij}},$$

(2) $$\sigma =2{\left(\frac{\sigma_{i}^{6}+\sigma_{j}^{6}}{2}\right)}^{1/6}$$

(3) $$\epsilon =\sqrt[{2}]{\epsilon_{i}\epsilon_{j}}\frac{\sigma_{i}^{3}\sigma_{j}^{3}}{\sigma_{i}^{6}+\sigma_{j}^{6}}$$

where $r_{ij}$ represents the charges, $q_{i}$ and $q_{j}$ are the separation distances from two force centers, $\epsilon_{0}$ is the relative permittivity, σ denotes the zero-interaction potential force distance, and ϵ is the highest amplitude. 

## 4. Conclusions

In this study, the adsorption capacities of H_2_, CO_2_, and CH_4_ were modeled on four kerogen structures of varying maturities under a wide range of pressures and temperatures. The adsorption capability of organic-rich matter (kerogen) improved with increasing pressure and decreased with temperature, regardless of kerogen maturity and gas type. The maximum adsorption was 10.6 mmol/g for CO_2_, 7.5 mmol/g for CH_4_, and 3.7 mmol/g for H_2_ in overmature II-D kerogen at 20 MPa and 323 K. Furthermore, the adsorption capacity was directly associated with thermal maturity, carbon content, and porosity at a certain pressure and temperature, i.e., II-D presented the highest adsorption, while all kerogens displayed mainly type I behavior for all gases. For all kerogens, adsorption followed the trend CO_2_ > CH_4_ > H_2_, attributed to the larger CO_2_ molecular size, which increased its affinity toward the kerogen. Furthermore, the available information from elementary analysis in terms of the pore size distribution was consistent with the adsorption trends. The porosity of kerogens and adsorption capacities of gases followed the order II-D > II-C > II-B > II-A, showing a direct correlation between adsorption capacity and kerogen porosity. Generally, overmature kerogen offered the maximum adsorption capacity at the maximum pressure and minimum temperature. 

These findings, therefore, contribute to the preliminary datasets of organic-rich shale reservoirs in terms of kerogen adsorption affinity towards different gases and related logical intellection of adsorption mechanisms in kerogens. In summary, the findings suggest that mature shale formations with relatively high pressure and low temperature are highly suitable for gas storage. These results are also important to understand the potential of unconventional shale for the competitive potential of CO_2_ and H_2_ storage and thus contribute to strategies for carbon emission/hydrogen economy. In addition, this work can be extended in the presence of brine phase for its effect on adsorption.

## Figures and Tables

**Table 1:** Trend of adsorption in four kerogen structures with temperature increment at a fixed pressure.

Structure	Percentage of Decline in Adsorption from 323 K to 423 K		
	CO_2_	CH_4_	H_2_
II-A	↓77%	↓77%	↓75%
II-B	↓65%	↓65%	↓75%
II-C	↓77%	↓62%	↓74%
II-D	↓77%	↓64%	↓73%

**Table 2:** Details of the kerogen units used in this investigation [91,93].

Kerogen Type	Chemical Formula	H/C	O/C	Density of Final Configuration, g/cm^3^	Maturity Level	Ø
IIA	C_252_H_294_O_24_N_6_S_3_	1.17	0.095	1.126	Immature	0.056
IIB	C_234_H_263_O_14_N_5_S_2_	1.12	0.06	1.103	Top of Oil Window	0.073
IIC	C_242_H_219_O_13_N_5_S_2_	0.91	0.054	1.168	Middle/End of Oil Window	0.075
IID	C_175_H_102_O_9_N_4_S_2_	0.58	0.051	1.240	Overmature	0.144

## Data Availability

Not applicable.
